# Decoupled dynamic magnetic field measurements improves diffusion-weighted magnetic resonance images

**DOI:** 10.1038/s41598-017-11138-8

**Published:** 2017-09-14

**Authors:** Ying-Hua Chu, Yi-Cheng Hsu, Fa-Hsuan Lin

**Affiliations:** 1 Institute of Biomedical Engineering, National Taiwan University, Taipei, Taiwan; 2 Department of Neuroscience and Biomedical Engineering, Aalto University, Espoo, Finland

## Abstract

Field probes are miniature receiver coils with localized NMR-active samples inside. They are useful in monitoring magnetic field. This information can be used to improve magnetic resonance image quality. While field probes are coupled to each other marginally in most applications, this coupling can cause incorrect resonance frequency estimates and image reconstruction errors. Here, we propose a method to reduce the coupling between field probes in order to improve the accuracy of magnetic field estimation. An asymmetric sensitivity matrix describing the coupling between channels of field probes and NMR active droplets within field probes was empirically measured. Localized signal originating from each probe was derived from the product of the inverse of the sensitivity matrix and the coupled probe measurements. This method was used to estimate maps of dynamic magnetic fields in diffusion weighted MRI. The estimated fields using decoupled probe measurement led to images more robust to eddy currents caused by diffusion sensitivity gradients along different directions.

## Introduction

Magnetic field probes consist of micro radio-frequency (RF) coils with enclosed nuclear magnetic resonance (NMR)-active droplets^1^. They are useful in estimating spatiotemporal magnetic field distributions in MRI experiments^1–4^. Previous studies demonstrated that field probes can be used to measure the *k*-space trajectory^2, 3^, estimate magnetic fields generated by heart beating and breathing^5, 6^, track motion^7^, correct artifacts in diffusion weighed imaging^8^, and characterize gradient system^9^. To estimate a magnetic field map, we need to use multiple field probes, each of which should be only sensitive to the magnetic field at the close proximity of a probe. To obtain such localized probe measurements, the coupling between probes needs to be minimized. Imperfect decoupling between probes causes systematic mixing of NMR signals from probes at different locations. As the magnetic field mapping is directly built upon the association between physical locations of individual probes and their NMR signals, NMR signal mixing among probes causes incorrect signal-location association and ultimately erroneous estimates of magnetic field distributions. Minimizing the signal mixing (good decoupling) has been suggested as the key to obtain high quality images using a multi-channel receiver coil array^10^. This can be done by appropriate overlapping between neighboring receiver coils, adding cable traps^11^, and using low input impedance pre-amplifiers^12^. The problem of residual RF coupling between probes has previously been reported^5^. However, to the best of our knowledge, the effect of coupled field probes and the strategy to decouple field probes have not been systematically studied.

Practically, the coupling in a field probe array is smaller than that in a receiver coil array because: *i*) the micro RF coil in each probe has a much smaller size, and *ii*) the separation between field probes are larger (considering the relative distance in terms of coil separation and coil size). However, probe coupling still exists due to parasitic capacitance to the common ground or cross-talk between transmission lines connected to probes^1, 13, 14^. In fact, the coupling can originate from any location along the RF pathway, including RF cabinet, coaxial cables, and circuit boards.

To explain how the coupled signal between probes affect the magnetic field estimation, in this paper, we mathematically described how the coupling among probes causes oscillatory instantaneous frequency estimates at each probe. Such oscillation can lead to erroneous magnetic field estimates and inaccurate image reconstruction. To decouple field probe measurements, inspired by the sensitivity-encoded MRI^15^, we propose a decoupling method using a sensitivity matrix to characterize the coupling between channels of field probes and NMR-active droplets within receive-only field probes. This method allows us to separate the measured field probe signal into the component originating from its own and the component originating from other probes. With the explicit description on how these two components are mixed using an empirically measured sensitivity matrix, we can minimize the probe coupling by multiplying the inversion of this sensitivity matrix to coupled probe measurements in order to obtain decoupled probe signals. The performance of this method was first demonstrated by reducing the erroneously estimated oscillatory magnetic field when a time-invariant gradient field was applied. We then applied probe decoupling to diffusion weighted images to correct eddy current artifacts. Note that applying diffusion sensitive gradients along different directions leads to different eddy currents. Consequently, differently distorted images are typically generated when the diffusion weighted gradients in different directions are used. However, with the magnetic field estimated from decoupled probes, effects of eddy currents can be accurately accounted for in the reconstructed images, which show more invariant structure across measurements using diffusion weighted gradients in different directions.

## Theory

### Behaviors of coupled field probes

To study the effect of probe coupling, we started from the simplest case, where a finite coupling exists between two probes such that the micro RF coil in one probe (probe 1) can receive NMR signals from the NMR-active droplets from both itself (probe 1) and the other probe (probe 2). The NMR signals from probes 1 and 2 oscillate at frequency *ω*_1_ and *ω*_2 _with signal strength *s*_1_ and *s*_2_, respectively. *η* is the coupling strength between two micro RF coils. The received signal at probe 1 is:

1 $${s}_{1}{e}^{i{\omega }_{1}t}+\eta {s}_{2}{e}^{i{\omega }_{2}t}={e}^{i{\omega }_{1}t}({s}_{1}+\eta {s}_{2}{e}^{i({\omega }_{2}-{\omega }_{1})t}).$$

Its instantaneous phase *ϕ*(t) of the received signal is

2 $${\rm{\varphi }}({\rm{t}})={\omega }_{1}t+\arctan (\frac{\sin (({\omega }_{2}-{\omega }_{1})t)}{\frac{{s}_{1}}{\eta {s}_{2}}+\,\cos (({\omega }_{2}-{\omega }_{1})t)}).$$

The instantaneous frequency estimated from the temporal derivative of the instantaneous phase is:

3 $$\frac{{\rm{d}}{\rm{\varphi }}({\rm{t}})}{{\rm{dt}}}={\omega }_{1}+\tfrac{1}{1+\tfrac{{\sin }^{2}(({\omega }_{2}-{\omega }_{1})t)}{{(\tfrac{{s}_{1}}{\eta {s}_{2}}+\cos (({\omega }_{2}-{\omega }_{1})t))}^{2}}}({\omega }_{2}-{\omega }_{1})\tfrac{\cos (({\omega }_{2}-{\omega }_{1})t)(\tfrac{{s}_{1}}{\eta {s}_{2}}+\,\cos (({\omega }_{2}-{\omega }_{1})t))+{\sin }^{2}(({\omega }_{2}-{\omega }_{1})t)}{{(\tfrac{{s}_{1}}{\eta {s}_{2}}+\cos (({\omega }_{2}-{\omega }_{1})t))}^{2}}.$$

Considering the probe coupling *η* is relatively weak, we assume that $\frac{{s}_{1}}{\eta {s}_{2}}\gg 1$. Accordingly, the instantaneous frequency is approximated as:

4 $$\frac{{\rm{d}}{\rm{\varphi }}({\rm{t}})}{{\rm{dt}}}\approx {\omega }_{1}+({\omega }_{2}-{\omega }_{1})\frac{\eta {s}_{2}}{{s}_{1}}\,\cos (({\omega }_{2}-{\omega }_{1}){t}).$$

This shows that the instantaneous frequency estimates will oscillate at the frequency *ω*_2_ − *ω*_1_ when two probes are coupled. Moreover, the effect of coupling $(({\omega }_{2}-{\omega }_{1})\frac{\eta {s}_{2}}{{s}_{1}})\,\,$is linearly proportional to the difference between two probe frequency (*ω*_2_ −* ω*_1_) and the coupling strength and the signal strength ratio $\frac{\eta {s}_{2}}{{s}_{1}}$. However, this oscillation can become negligible if the coupling is greatly reduced (*η* ≪ 1).

While we only derived the behavior of two coupled probes, the coupling between many probes can also be derived accordingly to show that the coupling between probes causes oscillation in the instantaneous frequency estimate at each probe. The instantaneous frequency of the NMR signal from a probe within a probe array with coupling among them is approximated as:

5 $${\omega }_{1}+\sum _{i=2}^{n}({\omega }_{i}-{\omega }_{1})\frac{{s}_{i}{\eta }_{i}}{{s}_{1}}\,\cos (({\omega }_{i}-{\omega }_{1}){t}).$$

where *ω*_*i*_, *s*_*i*_ and *η*_*i*_ denote precession frequency, signal strength, and coupling strength of probe *i*, respectively. This oscillatory instantaneous frequency in turn leads to oscillatory magnetic field estimate. Image correction based on these magnetic field estimates are thus incorrect. Taken together, decoupled probe measurements should provide more accurate estimates of local magnetic field strength to facilitate more accurate image reconstruction.

### Field probe decoupling

Here we described the procedure to decouple probes empirically. The location for the *i* ^th^ probe (1 ≤ *i* ≤ *n*) was denoted by the coordinates **r**_*i*_. *n* denotes the total number of probes. **r**_*i*_ can be estimated from the image reconstructed by data received at the micro-RF coil of the *i* ^th^ probe *C*_*i*_:

6 $${{\bf{r}}}_{i}={{\rm{argmax}}}_{{\boldsymbol{r}}}abs({C}_{i}({\bf{r}})).$$

After estimating locations of all probes, a sensitivity matrix **S**, where the element *S*_*ij*_ denotes the coupled NMR signal to field probe *i* from field probe *j*, can be constructed:

7 $${S}_{ij}={C}_{i}({{\bf{r}}}_{j}).$$

Note that the **S** matrix is neither a noise covariance matrix nor symmetric. The *ij* ^th^ component of the **S** matrix denotes the sensitivity of the receiving channel of the *i* ^th^ field probe to the droplet at the *j* ^th^ field probe. The sensitivity matrix **S** is not a symmetric matrix in general, because there is no reason to constrain that the sensitivity of the receiving channel of the *j* ^th^ field probe to the droplet of *i* ^th^ field probe is identical to the sensitivity of the receiving channel of the *i* ^th^ field probe to the droplet of *j* ^th^ field probe. The signals originating from droplets of field probes can be recovered by:

8 $$p={{\bf{S}}}^{-1}\tilde{p},$$

where *p* was a column vector denoting the decoupled probe signals. $\tilde{p}$ was a column vector denoting measured probe signals.

In this study, we used a probe array with 24 probes distributed over a plane (see Method below). We acquired gradient echo (GRE) images covering all field probes (flip angle = 25°, resolution = 0.5 mm × 0.5 mm, TE/TR = 10/100 ms) to estimate the sensitivity matrix **S**. Note that the image resolution was set after considering the size of the droplet within each probe, the required accuracy of probe localization, and Gibb’s ringing artifacts.

### Dynamic magnetic field estimation

After field probe decoupling, we dynamically estimated the local magnetic field by the phase change between two consecutively acquired NMR signal *p*_*j*_(*t*_*i*+1_) and *p*_*j*_(*t*_*i*_) at probe *j*:

9 $${b}_{j}({t}_{i})=\frac{\text{arg}(\frac{{p}_{j}({t}_{i+1})}{{p}_{j}({t}_{i})})}{2\pi \gamma ({t}_{i+1}-{t}_{i})},$$

where *t*_*i*_ denotes the time of acquiring the *i* ^th^ sample, *γ* is the gyromagnetic ratio, and *b*_*j*_ denotes the magnetic field estimate at probe *j*. The function *arg* gives the polar angle of a complex number. The dynamic magnetic field map at the imaging plane was estimated by fitting *b*_*j*_(*t*_*i*_), 1 ≤ *j* ≤ *n*, to a 2D spatial polynomial. Specifically, coefficients for the polynomial were estimated by the following optimization problem:

10 $${{\rm{a}}}_{kl}({t}_{i})=argmi{n}_{{\alpha }_{kl}}\sum _{j}({b}_{j}({t}_{i})-\sum _{k=0}^{q-l}\sum _{l=0}^{q}{\alpha }_{kl}{x}_{j}^{k}{y}_{j}^{l}{)}^{2},0\le k\le q-l,\,0\le l\le q,$$

where *q* denotes the highest order of the spatial polynomial used in the estimation. In this study, we used *q* = 4 to allow a large degree of freedom in characterizing the magnetic field distribution using 24 probes. The estimated *q* ^th^-order magnetic field map at acquisition time point *t*_*i*_ is:

11 $$\sum _{k=0}^{q-l}\sum _{l=0}^{q}{a}_{kl}({t}_{i}){x}^{k}{y}^{l}.$$

## Results

### Simulation studies of signal coupling in the gradient waveform, magnetic field estimate

**Figure 1:**
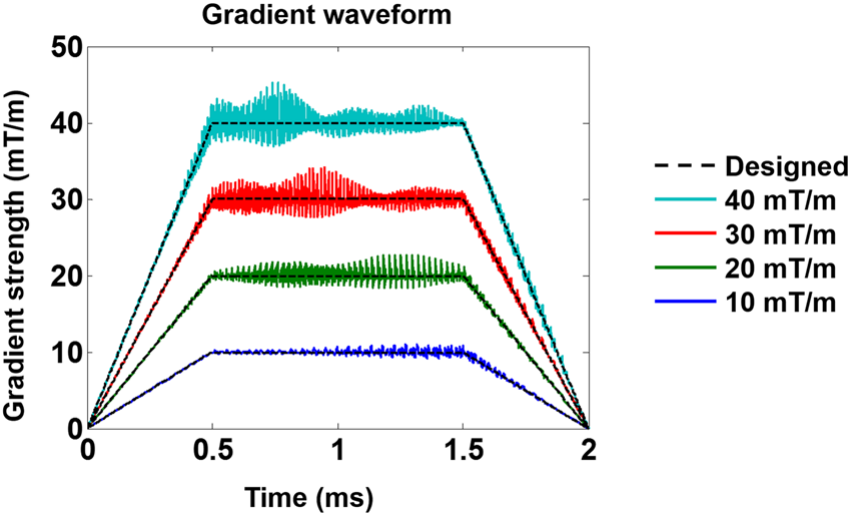
The estimated gradient waveforms of one EPI readout at gradient strengths of 10, 20, 30, and 40 mT/m. The dotted black lines show the designed gradient waveforms.

Probe coupling can affect the estimated magnetic field. Such effects can further propagate to gradient waveform, and trajectory estimates. Consider a theoretical case, where probes are fully coupled. In this case, all probes provide the same measurements. Consequently, only the 0^th^-order (constant) magnetic field can be estimated with zero effect on the gradient waveform and trajectory estimation. To examine how probe coupling affects the magnetic field, gradient waveform, and trajectory estimates in more practical scenarios, we simulated EPI gradient waveform estimated with zero coupling (the ideal case) and with empirically measured coupling between probes. Fig. 1 shows the estimated gradient waveforms of one EPI readout at different gradient strengths. We found that the higher the gradient strength, the larger oscillation in the estimated gradient waveform. Because a higher gradient strength results in a larger difference in the precessing frequency between coupled probes. And a larger difference in the precessing frequency causes a larger oscillation in the estimated magnetic field in one probe (Eq. [4]), which in turn leads to a larger oscillation in the estimated gradient waveform. Table 1 lists that the mean error in magnetic field estimates increased as the gradient strength increased. This error also increased as a higher order polynomial was used, presumably because the fitting error scaled with the number of parameters to be fitted. The mean error in accumulated phase estimates also increased as the gradient strength increased.

**Table 1:** (A) The mean errors in the magnetic field estimates fitted by various orders (1 to 4) of spatial polynomials over the entire EPI gradient waveform at maximal gradient amplitudes of 10, 20, 30 and 40 mT/m. (B) The mean errors in the accumulated phase estimates fitted by various orders (1 to 4) of spatial polynomials over the entire EPI gradient waveform at maximal gradient amplitudes of 10, 20, 30 and 40 mT/m.

Order of spatial polynomials	1	2	3	4
Gradient strength				
**(A) Error in the magnetic field strength estimates**				
10 mT/m	0.018	0.026	0.033	0.040
20 mT/m	0.034	0.052	0.069	0.084
30 mT/m	0.046	0.069	0.092	0.111
40 mT/m	0.058	0.086	0.115	0.140
**(B) Errors in accumulated phase estimates**				
10 mT/m	0.038	0.041	0.044	0.044
20 mT/m	0.064	0.072	0.079	0.081
30 mT/m	0.088	0.096	0.102	0.105
40 mT/m	0.113	0.121	0.127	0.133

### Signals coupling in the gradient waveform, magnetic field estimates, and *k*-space trajectories using empirical data

**Figure 2:**
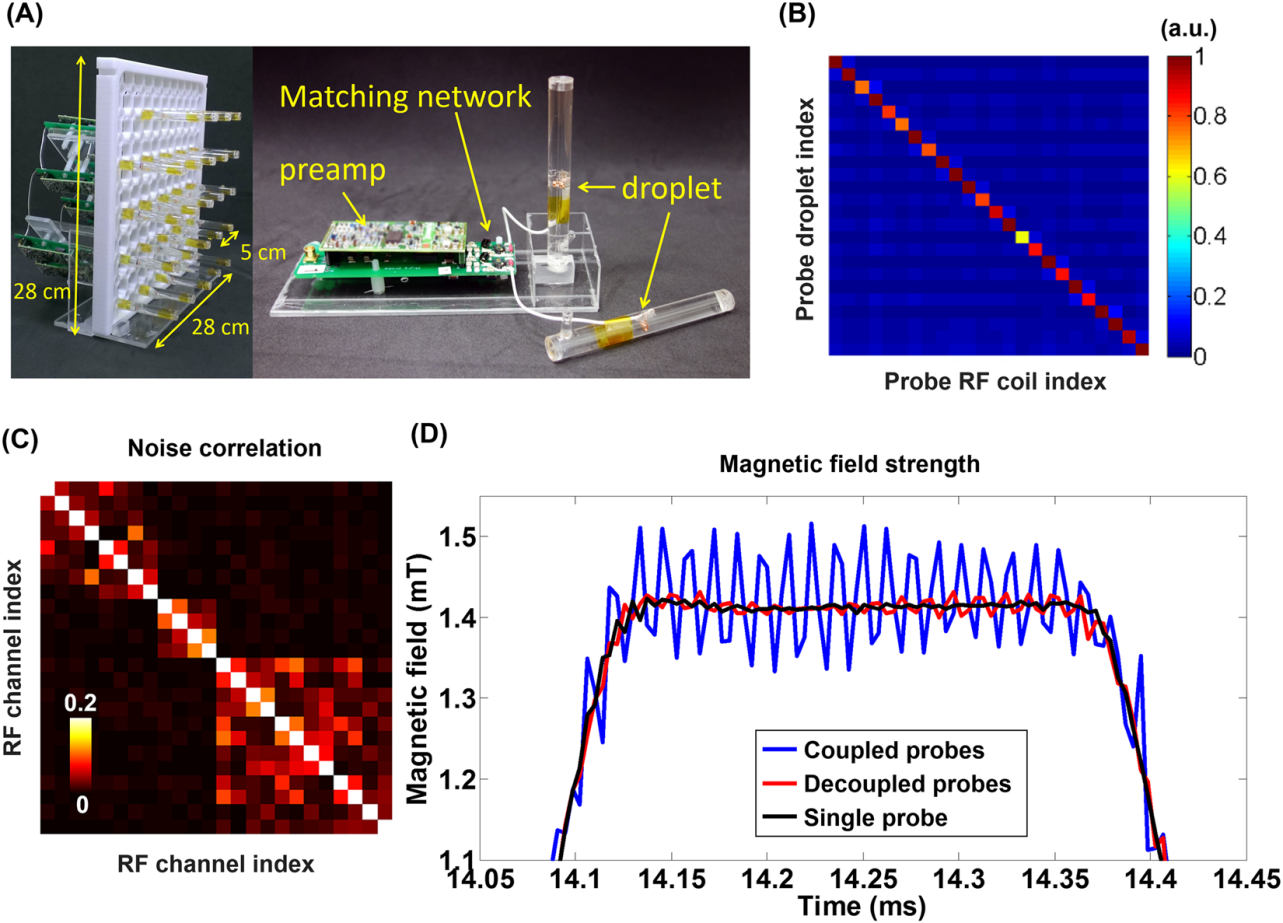
(**A**) Twenty-four field probes were arranged on a 2D grid structure. Each field probe consisted of a micro RF coil, water droplet, matching network and low-noise pre-amplifier. (**B**) The sensitivity matrix between channels of field probes and the droplets of field probes. (**C**) The noise correlation matrix of the RF system without any probe. (**D**) Dynamic magnetic field estimates at a single probe without other 23 probes (black), at a single probe with 24 coupled probes (blue), and at a single probe with 24 decoupled probes (red).

Figure 2B shows the sensitivity matrix **S** of the 24 channel probe array for probe decoupling. The mean absolute value of diagonal terms was 0.97 and the mean absolute value of off-diagonal terms was 0.025, which was 2.6% of the average of diagonal terms. Without any probe connected to the 24-channel RF system, there was still prominent coupling between channels (Fig. 2C; maximum off-diagonal elements = 0.11, and average off-diagonal elements = 0.015). This demonstrates that the system had non-negligible intrinsic coupling between RF channels. Note that this coupling matrix is different from the **S** matrix for probe decoupling. The estimated magnetic fields during one EPI readout from one single probe were shown in Fig. 2D, which includes three cases: i) with only this single probe and no other 23 probes. This case was considered the “best’ probe decoupling. ii) 24 coupled probes, and iii) 24 decoupled probes. During the plateau period of the readout, the magnetic field estimated from coupled field probes oscillated at a higher amplitude (peak-to-peak 0.14 mT) than from decoupled (peak-to-peak 0.02 mT) field probes. Probe decoupling suppressed the oscillation by 7 folds. This phenomenon can be explained by Eqs [4] and [5]. Note that, the oscillation observed here is not purely sinusoidal, potentially because this probe coupled to more than one probes. The average of the absolute value of the difference in the magnetic field estimated by coupled field probes and by a single probe was 0.063 mT. This difference became 0.010 mT after probe decoupling. Such 6-fold suppression of oscillation quantified the effect of probe decoupling.

**Figure 3:**
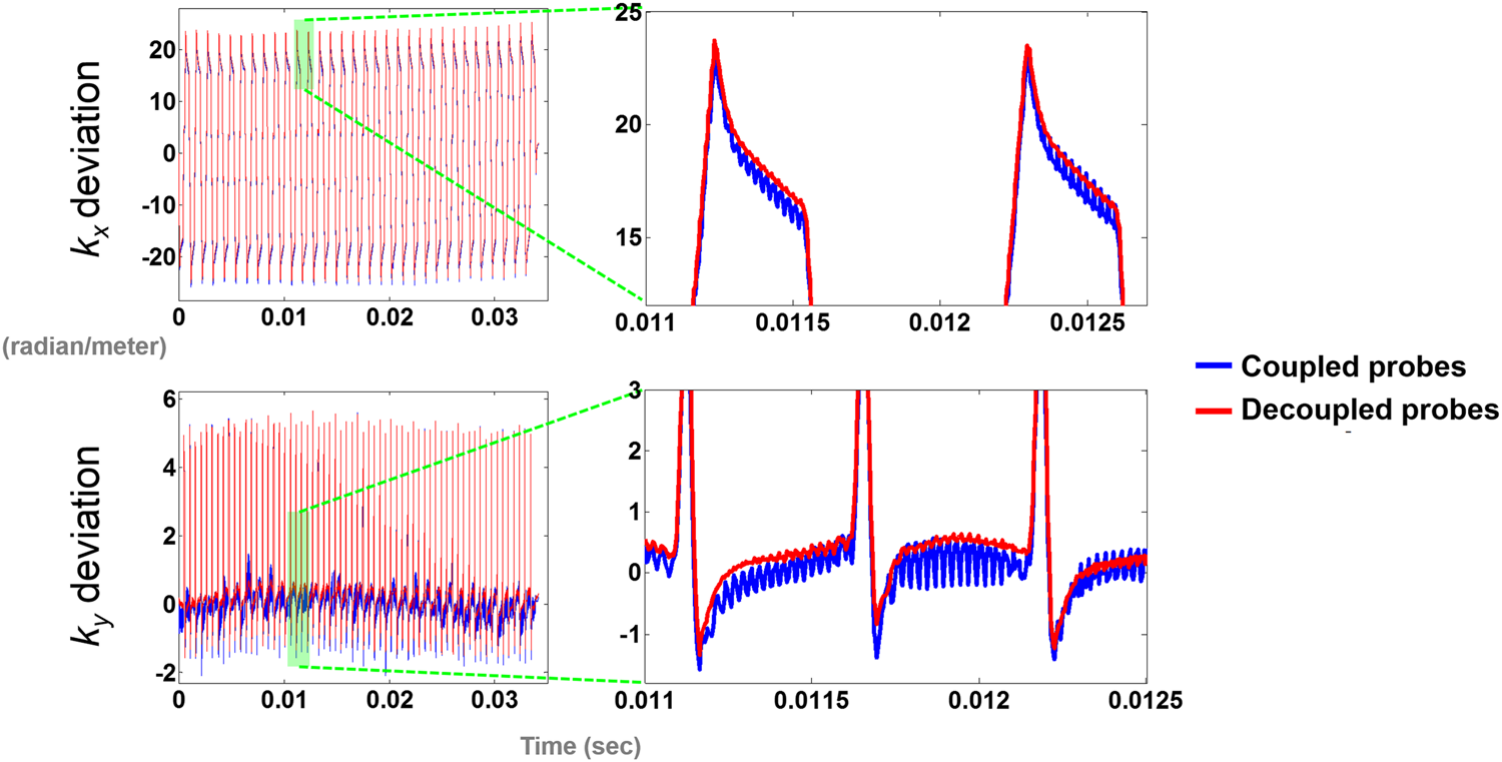
The *k*-space trajectory differences between the designed EPI trajectory and the estimated EPI trajectory without diffusion sensitive gradient using coupled and decoupled field probes.

The *k*-space trajectories deviation between the designed and the measured *k*_*x*_ and *k*_*y*_ EPI trajectories using coupled and decoupled field probes without diffusion sensitive gradient were shown in Fig. 3. These differences were caused by fast switching gradients in the EPI sequence, and illustrated the effect of eddy current in conventional EPI data collection and image reconstruction. The periodic oscillation was found with amplitude around 20 radian/m and 5 radian/m in the *k*_*x*_ and *k*_y_ axis, respectively. For an ideal gradient system, this deviation should be zero at all time instants. The *k*-space trajectories estimated from coupled field probes had clear high frequency oscillations. On the other hand, such oscillation has dramatically reduced in the *k*-space trajectory estimates using decoupled field probes. Such fast oscillations *k*-space trajectory is not physically likely, considering the gradient coil was driven in a much lower frequency.

**Figure 4:**
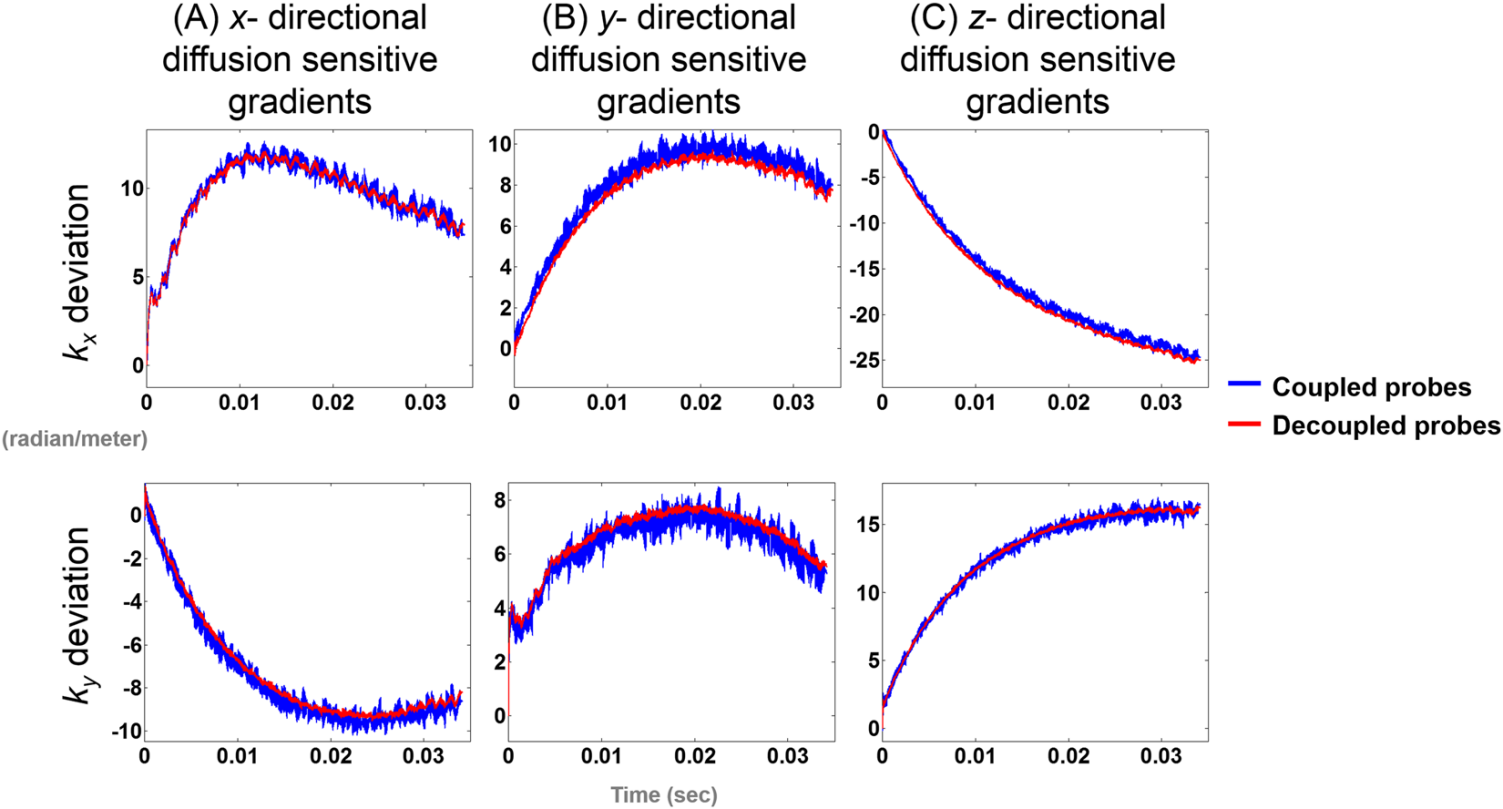
The *k*-space trajectory differences between the trajectory without diffusion sensitive gradient and the diffusion-weighted imaging trajectories with (**A**) *x*-directional diffusion sensitive gradient only, (**B**) *y*-directional diffusion sensitive gradient only, and (**C**) *z*-directional diffusion sensitive gradient only. Trajectories were estimated using coupled (blue traces) and decoupled (red traces) field probes.

The *k*-space trajectories estimated with and without applying diffusion sensitive gradient using coupled or decoupled probes in diffusion weighted imaging were shown in Fig. 4. Note that both trajectories were affected by the same eddy current caused by EPI acquisition gradient. Thus the difference between trajectory estimates was solely due to eddy current caused by diffusion sensitive gradients. Similar to Fig. 3, the measured trajectories using coupled field probes showed high frequency oscillations, which was physically unlikely. The eddy current caused by diffusion sensitive gradients led to a smooth shift of the *k*-space trajectory. Using decoupled probes, we estimated that this shift was no less than 8 radian/m and can be as strong as 25 radian/m.

### Diffusion weighted images

**Figure 5:**
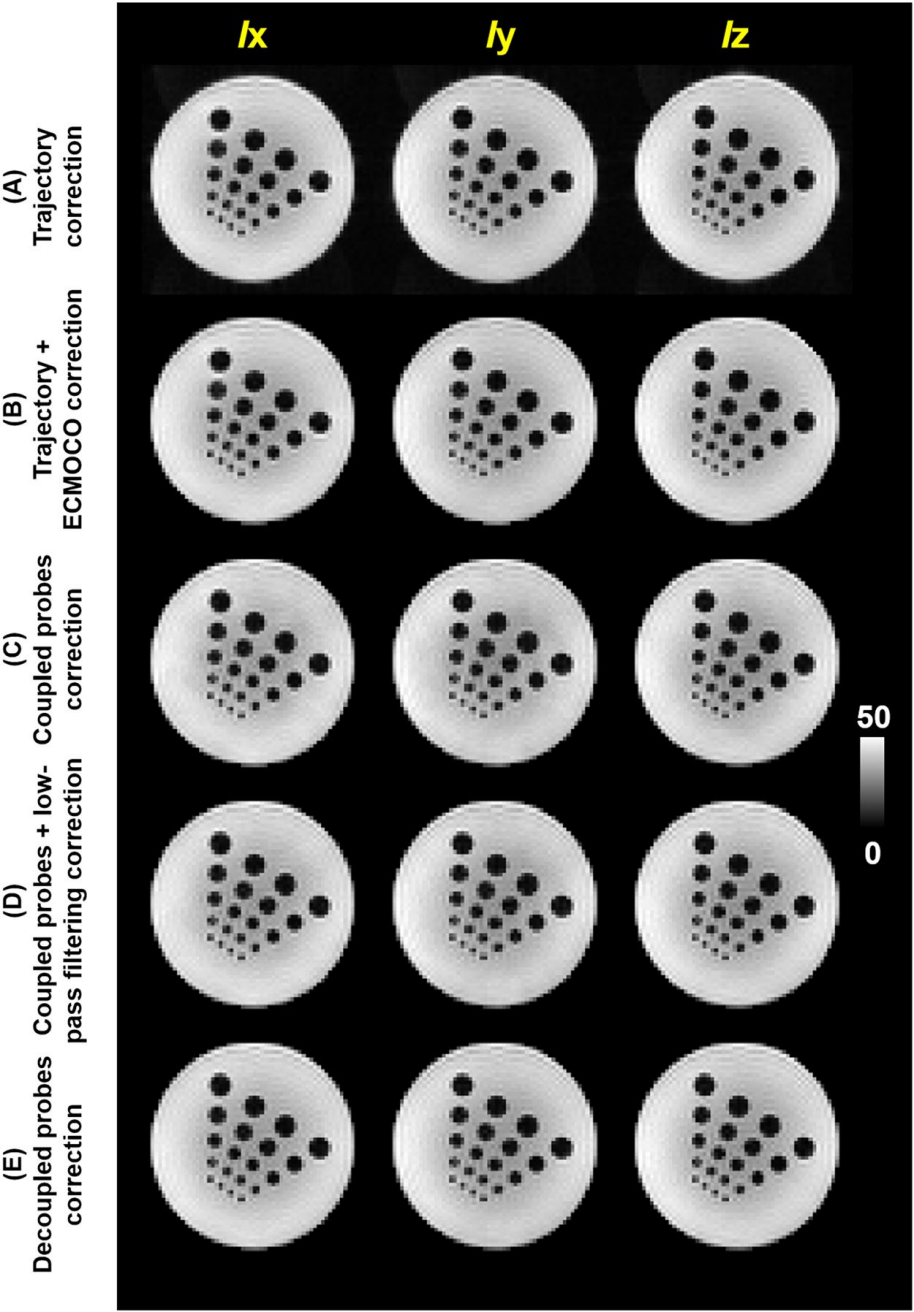
Diffusion weighted images in the unit of SNR with different directional diffusion sensitive gradients were reconstructed using (**A**) empirically measured trajectory, (**B**) empirically measured trajectory with ECMOCO post-processing, (**C**) coupled field probes, (**D**) coupled field probes with low-pass filtering, and (**E**) decoupled field probes.

The reconstructed diffusion weighted images *Ix*, *Iy*, and *Iz* with only the *x*-, *y*- and *z*- directional diffusion sensitive gradients, respectively, were shown in Fig. 5 in the unit of SNR. They were diffusion weighted images reconstructed with empirically measured trajectory, with empirically measured trajectory and post-processing, with coupled field probes, with coupled field probes and low-pass filtering, and with decoupled field probes. The images appeared visually similar.

**Figure 6:**
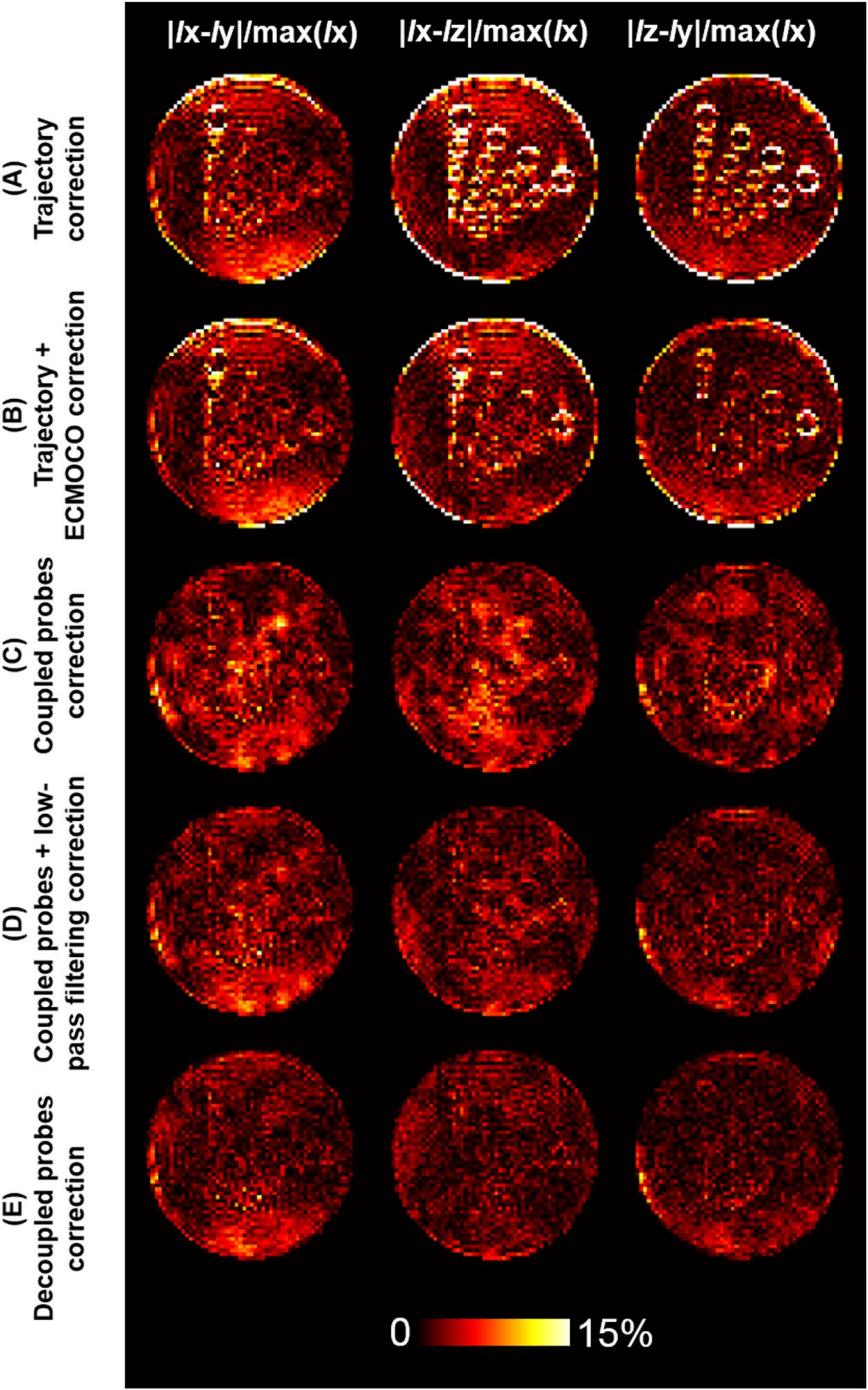
The difference images between diffusion weighted images with diffusion sensitive gradients along different directions using (**A**) empirically measured trajectory, (**B**) empirically measured trajectory with ECMOCO post-processing, (**C**) coupled field probes, (**D**) coupled field probes with low-pass filtering, and (**E**) decoupled field probes.

To better visualize the reconstruction errors using coupled or decoupled field probes, we took difference images between two diffusion weighted images with their diffusion sensitive gradients in two different directions (Fig. 6). Note that the difference images were divided by the highest value of the *I*x for visual inspection purpose. Ideally, the phantom has no structure sensitive to any particular direction. Thus such difference images should have zero pixel values everywhere. Quantitatively, mean differences were 3.36%, 4.81%, 3.86% for |*Ix* – *Iy*|/max(*Ix*), |*Ix* – *Iz*|/max(*Ix*) and |*Iz* – *Iy*|/max(*Ix*) using empirically measured trajectory, respectively. Mean differences became 3.18%, 3.33%, 2.71% for |*Ix* – *Iy*|/max(*Ix*), |*Ix* – *Iz*|/max(*Ix*) and |*Iz* – *Iy*|/max(*Ix*) after ECMOCO post-processing, respectively. Mean differences dropped to 2.83%, 2.77%, 2.26% using coupled field probes without low-pass filtering, and 2.58%, 2.06%, 1.83% using coupled field probes with low-pass filtering, respectively. Finally, using decoupled field probes, the mean differences were 2.17%, 1.77%, 1.59%, respectively. These differences were smaller than results from coupled probes with and without low-pass filtering. Note that during error quantification, the same mask was applied to all images.

**Figure 7:**
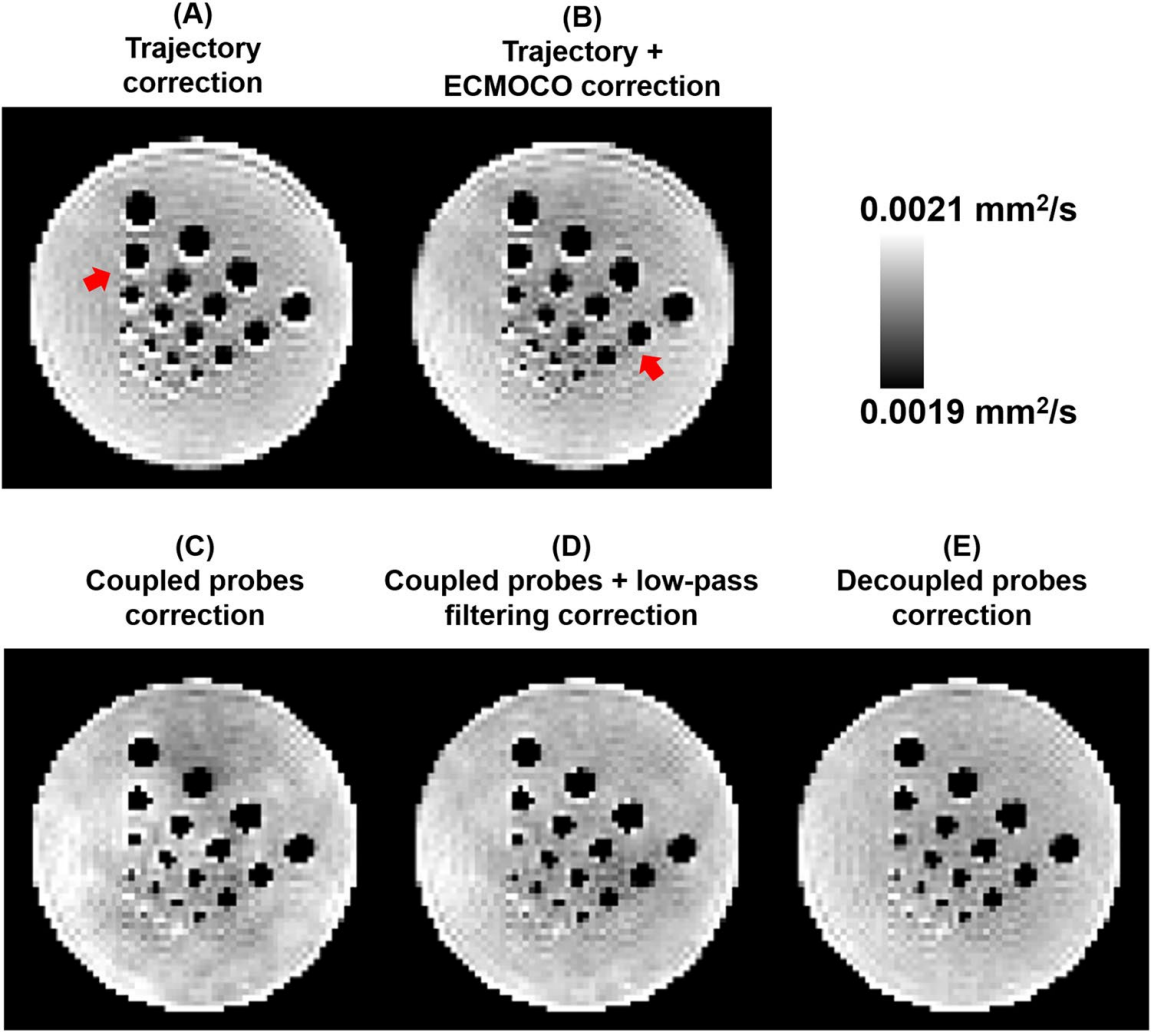
ADC maps estimated from (**A**) empirically measured trajectory, (**B**) empirically measured trajectory with ECMOCO post-processing, (**C**) coupled field probes, (**D**) coupled field probes with low-pass filtering, and (**E**) decoupled field probes. The red arrow in (**A**) indicates the artifact caused by inaccurate magnetic field estimates. The red arrow in (**B**) indicates the artifact reduced by ECMOCO correction.

Comparing to the uncorrected apparent diffusion coefficient (ADC) image (Fig. 7A), the ECMOCO-corrected ADC image reduced unlikely high ADC values at the boundary of tube structures inside the phantom (the red arrow in Fig. 7B). This suggested that the ADC images reconstructed from diffusion sensitive gradients along different directions were likely distorted due to the eddy current.

These artifacts were reduced when we reconstructed ADC images using the 4^th^-order correction (Fig. 7C–7E). The low-pass filtering can reduce the inhomogeneity caused by inaccurate magnetic field estimated from coupled field probes. However, the ADC map in Fig. 7E shows even more homogeneous ADC values using the magnetic field estimated from decoupled field probes. Together, we considered that correction using decoupled probes provided the best reconstruction. The standard deviations of ADC maps in Fig. 7A–E were 6.0 × 10^−5^, 4.9 × 10^−5^, 3.4 × 10^−5^, 3.2 × 10^−5^ and 3.1 × 10^−5^ mm^2^/s, respectively.

**Figure 8:**
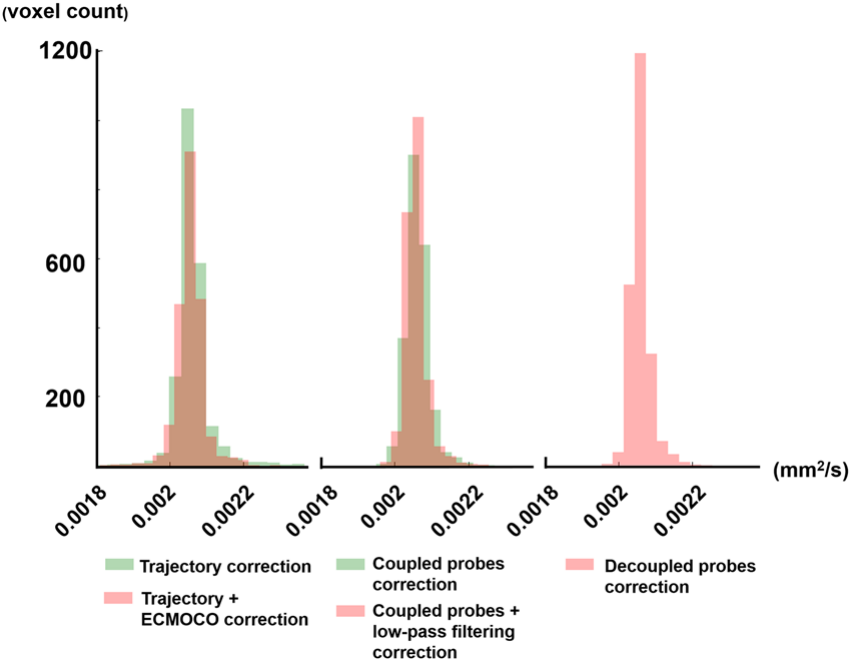
Histograms of ADC values estimated from the empirically measured trajectory, empirically measured trajectory with ECMOCO post-processing, coupled field probes, coupled field probes with low-pass filtering, and the decoupled field probes.

Lastly, we also calculated histograms of ADC maps reconstructed with different corrections. Note that the histogram from the reconstruction using decoupled probes shows the narrowest and the highest peak (Fig. 8), suggesting that the ADC values were most centrally distributed.

## Discussion

We derived the formula describing how the coupling between field probes results in oscillation in instantaneous frequency estimates. The amplitude of this oscillation is linearly proportional to the coupling strength, the difference of the precession frequency between two probes, and the ratio of their signal strengths. To overcome this coupling problem, we proposed a method to decouple field probes measurements and to improve the accuracy of dynamic magnetic field map estimation. We measured the sensitivity matrix of receiving channels of field probes, and used this information to decouple probe measurements. After this decoupling, we found that the oscillation of the estimated instantaneous frequency was reduced (Fig. 2D). The estimated *k*-space trajectory was smoother without noise-like fluctuations after probe decoupling (Figs 3 and 4). The difference image between diffusion weighted images using diffusion sensitive gradients along different direction was also reduced after probe decoupling (Fig. 6E). Compared with reconstructions using coupled field probe data, the mean image reconstruction error using decoupled field probes decreased from 2.6% to 1.8%. Furthermore, the ADC maps were more homogeneous when reconstructed using decoupled field probes (Fig. 7C,E). These results supported the value of probe decoupling to improve the dynamic field map estimation and to achieve more accurate image reconstruction with minimal eddy current artifacts.

The field probe coupling was not considered serious previously because cable traps can reduce the coupling^1^. Via inspection, our results also showed that images using information estimated from coupled and decoupled field probes were similar (Fig. 5C,E). Note that the exponents of spatial harmonics in the image encoding matrix were temporal integrals of the spatial encoding magnetic field, which was related to both magnetic field gradients and eddy currents. The oscillatory behavior of the measured magnetic field was consequently greatly reduced after such temporal integration. However, compared between images with different directionally sensitive diffusion weightings, we found that the image reconstruction error caused by eddy current from diffusion sensitive gradients was higher in average using coupled field probes (Fig. 6).

Aside from the demonstration of the decoupled probes in diffusion weighted images reported in this study, we hypothesize that probe decoupling will be crucial in, for example, characterizing the gradient performance using an impulse response function^9^. We expected that more accurate characterization can be obtained by using decoupled field probes than using coupled field probes. This hypothetical application will be explored in the near future.

In this study, our 24 field probes were distributed over a plane. This probe arrangement was different from the 16-probe array over a volume^16^. Our 24-probe planar array was more efficient in measuring high-order spatial distributions of the magnetic field in slice-selective imaging. For example, fitting a magnetic field over a plane to a 4^th^-order polynomial needs at least 15 probes on that plane, while characterizing the magnetic field over a plane with the accuracy affordable by 4^th^-order polynomials using spherical harmonics needs at least 25 probes distributed over the surface of a volume. Therefore, to achieve the same accuracy in slice-selective imaging, arranging probes over a plane is more efficient in describing the magnetic field than probes arranged over the surface of a volume.

Obviously one disadvantage of our planar array is that it only characterizes the magnetic field over one single plane. We may mechanically move the planar array to characterize the magnetic field over a volume. Alternatively, further development of a planar array into 3D, such as the 64-channel probe array^17^, can alleviate this tedious procedure.

In this study, we used GRE images with 0.5 mm × 0.5 mm in-plane resolution to estimate the sensitivity matrix. This spatial resolution was chosen after considering the size of the droplet within each probe, the required accuracy of probe localization, and Gibb’s ringing artifacts. We would recommend using a higher spatial resolution to avoid the potential confound of the ringing artifacts related to a wider point spread function in low-resolution images.

In this study, the location of a probe was estimated from the brightest voxel in an image (Eq. [6]). As this image was measured from coupled probes, the accuracy of the estimated probe locations can affect by probe coupling. However, this effect is rather small: considering 5 cm separation between probes, 0.5 mm spatial resolution in the image for probe location estimation, and the maximal coupling between probe of 0.1, the image pixel intensity at the probe location affected by coupling was only 0.2%. Therefore, we neglected the effect of coupled probes on probe location estimation in our study.

One alternative approach in estimating probe locations is to use constant gradient waveforms in three directions. However, to estimate the **S** matrix using constant gradient waveforms, a sufficiently long readout with a strong magnetic field gradient is required to separate signals from multiple probes, if no two probes generate the oscillatory NMR signal of the same frequency. Probe locations can be more efficiently estimated by 2D Fourier encoding.

Our probes in this study were receive-only. If probes can both transmit and receive NMR signals, we can measure the sensitivity matrix **S** more efficiently by transmitting RF in one probe and receiving the signal in the others. However, this requires multiple transmitters and the minimized coupling between transmitters.

Here we want to emphasize that the sensitivity matrix **S** in this study is essentially different from the noise correlation matrix among probes. This difference can be understood from parallel imaging, where coil sensitivity maps (as the sensitivity matrix **S** in this study) is completely different from the noise covariance matrix.

It is possible to use more robust regression methods in estimating the magnetic field using coupled or decoupled probes such as L1 regression. However, the stability and the order of the fitted magnetic field using other regressions depends on the probability distribution of the error in magnetic field estimate. This remains to be explored in the near future.

In the diffusion weighted imaging *k*-space trajectory estimation, we assumed that each trajectory started from the same *k*-space location (Fig. 4). However, there can be phases accumulated before probe signal readout. We ignored such phases in this study. As long as this phase within imaging voxels is small, this effect will only result a phase variation across the imaging plane without changing the apparent diffusion coefficient estimation.

Although residual RF coupling between probes can be reduced by a balanced transmission line design or using baluns/cable traps^1^, it has been addressed and mitigated recently by resolving the coupling matrix^5^. To overcome the limitation of the host RF system such as measurement timing/bandwidth and receiver specifications, previous studies used a customized stand-alone system which equipped with full RF transmitting/receiving system^1, 18^. However, most customized receiver coil arrays share the same RF system with the host MRI system. This RF system, which is difficult to modify, can introduce unwanted coupling between channels (Fig. 2C). Similar to our field probe array, the non-negligible coupling from cables between pre-amplifiers and the RF cabinet^13, 14^ cannot be avoided completely. To investigate the effect on magnetic field estimates from coupled field probes, we quantified and systematically analyzed the measurements difference between coupled and decoupled field probes. Results show that probe decoupling is important to provide accurate magnetic field estimation in practice.

In conclusion, we proposed a method of obtaining localized magnetic field estimates from field probes. We demonstrated the benefit of probe decoupling in diffusion weighted imaging in minimizing eddy current artifacts. Other MRI applications that can be benefitted by using probes to dynamically characterize magnetic field distributions, such as eddy current artifact correction, gradient coil performance measurements, and concomitant artifacts correction, should consider using decoupled probe data to improve the accuracy of magnetic field estimation.

## Method

### Field probe construction

A single field probe consisted of NMR-active droplet with susceptibility matching liquid, a micro RF coil, and a pre-amplifier. Here we chose water as the NMR signal source. Such a choice allowed high SNR, longer signal lifetime (longer *T*_2_ relaxation time), and direct signal processing using the same console for both probe processing and MRI experiments. A water droplet was held in the middle of a cylindrical glass capillary tube (1 mm inner diameter; 0.25 mm wall thickness), which was placed at the center of a cylindrical acrylic tube (1 cm inner diameter; 1 mm wall thickness; 9 cm length). Both ends of the water droplet and the acrylic tube were filled with susceptibility-matched liquid (FC40; 3 M, St. Paul, MN, USA)^1, 19^. The NMR signal was detected by a micro RF coil consisting of two 3-turn loops using 31 AWG copper wire. The water droplet was placed between two loops to detect the NMR signal. The coil was connected to a low-noise pre-amplifier integrated with a mixer (Siemens, Erlangen, Germany) through a 10-cm coaxial cable and a matching network. The matching network had a balanced circuit design and transformed the impedance to 50 Ω in order to obtain the lowest noise figure. The micro RF coil was tuned to 123.25 MHz and actively detuned by a PIN diode during RF transmission. Twenty-four field probes were arranged on a 5-by-5 3D-printed grid structure (PC-ISO: Fortus 400 mc, Stratasys, MN, USA) with 5 cm separation between field probes. Figure 2A shows the details of one probe and the 24-channel planar probe array. The coupling between RF channels was quantified by a noise correlation matrix, which was calculated from the imaging data collected by the RF system without any probe connected to it using a 2D gradient echo sequence without any RF transmission power (FOV = 250 × 250 mm^2^, slice thickness = 7 mm, TR = 8.6 ms, TE = 4 ms, flip angle = 0°, BW = 320 Hz/pixel, image matrix = 512 × 512 pixels).

### Simulation of probe coupling using EPI gradient waveform

To further understand how the finite coupling between probes affect the order of polynomial fits, gradient waveform, and trajectory estimates, we simulated EPI gradient waveform estimated with zero coupling (the ideal case) and with empirically measured coupling between probes.

In the simulation, we used EPI gradient waveform, which is composed of 64 trapezoidal gradient waveforms, with 500 us ramp time, 1000 us flat top time and maximal amplitude of 10, 20, 30, and 40 mT/m. The field probe array with the same **S** matrix and location as the empirical data were used in this simulation with sampling rate 1 MHz. The 1^st^ to 4^th^-order dynamic magnetic field map (*q* = 1, 2, 3, 4 in Eq. [11]) were estimated, and the estimation error was quantified as the mean magnetic field difference between the estimated magnetic field and the actual magnetic field at 24 probe locations over the entire EPI gradient waveform. Due to the fact that the accumulated phase error is directly related to the image reconstruction error, we also quantified the accumulated phase error as the mean difference between the estimated accumulated phase and the actual accumulated phase at 24 probe locations over entire EPI gradient waveform. Note that we did not measure the error of the gradient waveform or *k*-space trajectory, because both the gradient waveform and the *k*-space trajectory make more sense when the linear magnetic field (the 1^st^ order polynomial fit) is involved.

### Gradient waveform and *k*-space trajectories estimation of diffusion weighted imaging

The measurements were acquired on a 3 T MRI scanner (Skyra, Siemens Medical Solutions, Erlangen, Germany). The diffusion weighted imaging (TR = 6400 ms, TE = 75 ms, flip angle = 90°, *b* = 1000 s/mm^2^, 3 orthogonal diffusion sensitive gradient directions, FOV = 192 × 192 mm^2^, image matrix = 64 × 64, and slice thickness = 4 mm) was performed using a spin-echo diffusion sequence^20^ with EPI readouts. Note that, the probes were excited after turning off the diffusion sensitive gradients. This adjustment was necessary to minimize the dephasing of the magnetization of the NMR-active droplet inside the probe caused by diffusion gradients.

Three dynamic magnetic field waveforms during one EPI readout were plotted: 1) a localized dynamic magnetic field estimated at a single probe without other 23 probes, 2) a localized dynamic magnetic field estimated at a single probe with 24 coupled probes, and 3) a localized dynamic magnetic field estimated at a single probe with 24 decoupled probes.

We also measured *k*-space trajectories (with the constraint *q* = 1 in Eq. [10]) from coupled and decoupled field probes. The 2D *k*-space coordinates at time instant *t*_*τ*_ were

12 $$\begin{array}{c}{k}_{x}({t}_{\tau })=\sum _{l=1}^{\tau }{a}_{10}({t}_{l})\,\times \,2\pi \gamma ({t}_{l+1}-{t}_{l}),\\ {k}_{y}({t}_{\tau })=\sum _{l=1}^{\tau }{a}_{01}({t}_{l})\,\times \,2\pi \gamma ({t}_{l+1}-{t}_{l}).\end{array}$$

The trajectories difference between the designed and the measured trajectories without applying diffusion sensitive gradient were calculated. This difference trajectory was meant to characterize eddy current effects caused by fast switching gradients in EPI. The difference between two measured trajectories, with and without applying diffusion sensitive gradient, were also calculated. This difference trajectory was meant to characterize eddy current effects solely caused by the diffusion sensitive gradients.

### Diffusion weighted imaging

Images were acquired on a 3 T MRI scanner using a 20-channel head coil array (Skyra, Siemens Medical Solutions, Erlangen, Germany). The slice-selective diffusion weighted imaging was performed using a spin-echo diffusion sequence, where all imaging parameters were identical to the probe measurement without the excitation adjustment. The encoding matrix of the diffusion weighted images was created by four different kinds of dynamic magnetic field maps. Then, images were reconstructed using the conjugate gradient method^21^ includes higher-order fields in the reconstruction. Four different kinds of dynamic magnetic field maps in this study were: 1) the 4^th^-order dynamic magnetic field maps (*q* = 4 in Eq. [11]) estimated from coupled field probes, 2) the low-pass filtered (cut-off frequency = 20 kHz^9^) 4^th^-order dynamic magnetic field map estimated from coupled field probes, 3) the 4^th^-order dynamic magnetic field map estimated from decoupled field probes, and 4) the 1^st^-order dynamic magnetic field map estimated from decoupled probes (*q* = 1 in Eqs [10] and [12]). Note that all the 4^th^-order dynamic magnetic fields were separately estimated when each directionally sensitive diffusion gradient field was turned on and off. The 1^st^-order dynamic magnetic field was only estimated with all directionally sensitive diffusion gradients were off. This latter reconstruction was meant to represent the common practice of diffusion-weighted image reconstruction, where the reconstruction with empirically measured trajectory accounted for the gradient delays between even and odd echoes without considering effects caused by eddy currents generated by diffusion sensitive gradients. Additional images were obtained from the post-processing of the forth reconstruction method using the ECMOCO software in SPM^22^ to correct eddy current artifacts in a common diffusion processing pipeline.

Ideally, phantom images should change minimally when different diffusion sensitive gradients were applied, because the underlying structure provides no directional preference to proton diffusion. Accordingly, we calculated the difference image between two diffusion-weighted images with diffusion sensitive gradients applied to different directions in order to understand the performance of eddy current correction. We calculated the mean of the difference image as an index to estimate the error of the reconstructed image. Finally, we showed images of the estimated apparent diffusion coefficient (ADC) and their histograms.

## References

[1] De Zanche N, Barmet C, Nordmeyer-Massner JA, Pruessmann KP (2008). NMR probes for measuring magnetic fields and field dynamics in MR systems. Magn Reson Med.

[2] Barmet C, De Zanche N, Pruessmann KP (2008). Spatiotemporal magnetic field monitoring for MR. Magn Reson Med.

[3] Sipila P, Greding S, Wachutka G, Wiesinger F (2011). H-2 Transmit-Receive NMR Probes for Magnetic Field Monitoring in MRI. Magn Reson Med.

[4] Barmet C, De Zanche N, Wilm BJ, Pruessmann KP (2009). A Transmit/Receive System for Magnetic Field Monitoring of *In Vivo* MRI. Magn Reson Med.

[5] Vannesjo SJ (2015). Retrospective Correction of Physiological Field Fluctuations in High-Field Brain MRI Using Concurrent Field Monitoring. Magn Reson Med.

[6] Gross S (2016). Dynamic nuclear magnetic resonance field sensing with part-per-trillion resolution. Nature communications.

[7] Ooi MB, Krueger S, Thomas WJ, Swaminathan SV, Brown TR (2009). Prospective Real-Time Correction for Arbitrary Head Motion Using Active Markers. Magn Reson Med.

[8] Wilm BJ (2015). Diffusion MRI with concurrent magnetic field monitoring. Magn Reson Med.

[9] Vannesjo SJ (2013). Gradient system characterization by impulse response measurements with a dynamic field camera. Magn Reson Med.

[10] Wiggins GC (2006). 32‐channel 3 tesla receive‐only phased‐array head coil with soccer‐ball element geometry. Magn Reson Med.

[11] Seeber DA, Jevtic I, Menon A (2004). Floating shield current suppression trap. Concept Magn Reson B.

[12] Roemer PB, Edelstein WA, Hayes CE, Souza SP, Mueller OM (1990). The NMR Phased Array. Magn Reson Med.

[13] Chen, C.-n. & Hoult, D. I. *Biomedical magnetic resonance technology*. Vol. 100 (Adam Hilger Bristol, 1989).

[14] Vaughan, J. T. & Griffiths, J. R. *RF coils for MRI*. (John Wiley & Sons, 2012).

[15] Pruessmann KP, Weiger M, Scheidegger MB, Boesiger P (1999). SENSE: sensitivity encoding for fast MRI. Magn Reson Med.

[16] Duerst Y (2015). Real-time feedback for spatiotemporal field stabilization in MR systems. Magn Reson Med.

[17] Tountcheva, V. *et al*. A 64channel receive-only field camera for eddy current and trajectory calibration. *In Proceedings of the 20th Annual Meeting ISMRM, Melbourne, Australia*, 701 (2012).

[18] Dietrich BE (2016). A field camera for MR sequence monitoring and system analysis. Magn Reson Med.

[19] Subramanian R, Lam MM, Webb AG (1998). RF microcoil design for practical NMR of mass-limited samples. J Magn Reson.

[20] Stejskal, E. O. & Tanner, J. E. Spin Diffusion Measurements: Spin Echoes in the Presence of a Time-Dependent Field Gradient. *J Chem Phys* **42**, 288- + , doi:10.1063/1.1695690 (1965).

[21] Wilm BJ, Barmet C, Pavan M, Pruessmann KP (2011). Higher order reconstruction for MRI in the presence of spatiotemporal field perturbations. Magn Reson Med.

[22] Mohammadi S, Möller HE, Kugel H, Müller DK, Deppe M (2010). Correcting eddy current and motion effects by affine whole‐brain registrations: Evaluation of three‐dimensional distortions and comparison with slicewise correction. Magnetic Resonance in Medicine.

